# Effects of short-term exposure to particulate matter air pollution on cognitive performance

**DOI:** 10.1038/s41598-019-44561-0

**Published:** 2019-06-03

**Authors:** M. A. Shehab, F. D. Pope

**Affiliations:** School of Geography, Earth and Environmental Sciences, University of Birmingham, Edgbaston, Birmingham B15 2TT UK

**Keywords:** Environmental impact, Neurological manifestations

## Abstract

This paper assesses the effect of short-term exposure to particulate matter (PM) air pollution on human cognitive performance via a double cross over experimental design. Two distinct experiments were performed, both of which exposed subjects to low and high concentrations of PM. Firstly, subjects completed a series of cognitive tests after being exposed to low ambient indoor PM concentrations and elevated PM concentrations generated via candle burning, which is a well-known source of PM. Secondly, a different cohort underwent cognitive tests after being exposed to low ambient indoor PM concentrations and elevated ambient outdoor PM concentrations via commuting on or next to roads. Three tests were used to assess cognitive performance: Mini-Mental State Examination (MMSE), the Stroop Color and Word test, and Ruff 2 & 7 test. The results from the MMSE test showed a statistically robust decline in cognitive function after exposure to both the candle burning and outdoor commuting compared to ambient indoor conditions. The similarity in the results between the two experiments suggests that PM exposure is the cause of the short-term cognitive decline observed in both. The outdoor commuting experiment also showed a statistically significant short-term cognitive decline in automatic detection speed from the Ruff 2 and 7 selective attention test. The other cognitive tests, for both the candle and commuting experiments, showed no statistically significant difference between the high and low PM exposure conditions. The findings from this study are potentially far reaching; they suggest that elevated PM pollution levels significantly affect short term cognition. This implies average human cognitive ability will vary from city to city and country to country as a function of PM air pollution exposure.

## Introduction

Recent evidence has shown that particulate matter (PM) air pollution can have adverse effects on the mature nervous system in adults^1–4^. However, the association between the effect of air pollution and cognitive functions remains largely unexplored^5–7^.

To maintain and protect air quality and physical human health, the World Health Organization (WHO) provides guidelines for threshold pollutant concentration limits^8^. In particular, the WHO recommend that daily and annual limits of fine PM (PM_2.5_, which has a diameter less than or equal to 2.5 μm) do not exceed 25 and 50 µg/m^3^, respectively, to limit negative effects on human mortality and morbidity. However, there exist no specific recommendations for safe PM limits with respect to cognition and mental health.

Urban commuting, including walking, cycling, and different forms of motorized transportation (e.g. train, bus, private car) is a major source of personal exposure to PM_2.5_ because commuters are located close to the emissions from vehicles, which are often the major source of urban PM^9^. In addition to PM, vehicular sources emit other pollutants into the atmosphere, including hydrocarbons (HC), nitrogen oxides (NOx), carbon monoxide (CO), and sulphur dioxide (SO_2_)^10^. In 2015, air pollution caused approximately nine million premature deaths worldwide^11^; PM_2.5_ is responsible for approximately 4.5 million of these deaths, which makes it the fifth highest ranked risk factor for global deaths^12^. For example, in the UK every year, air pollution causes approximately 40,000 premature deaths, about half of which are associated with the pollutants emitted from motorized transport^13^. Hence, people who commute on busy roads have elevated likelihoods of adverse health effects^14^, which can lead to enhanced morbidity and mortality^15^.

Another source of personal exposure to fine PM is candle burning, which produces soot, and other types of PM^16^. Lighting candles can elevate indoor PM concentrations fivefold^12^, and when inhaled by indiviuals, can cause cardiopulmonary problems^17–22^. Candles are used in many situations, sometimes on a daily basis, for example for lighting, religious or spiritual purposes and relaxing. In addition to the importance of candles as a potential indoor pollution source, they also provide an easily controlled source of PM for indoor exposure experiments.

The six cognitive domains of the brain that can be assessed using different cognitive tests are: Visual-Spatial, Executive Function, Verbal Fluency, Memory, Attention, and Orientation^23^. Summaries of the six cognitive domains are provided in the Supplementary Material.

This research hypothesizes that short term personal exposure to PM adversely affects cognitive performance. The hypothesis is tested with two distinct experiments: Experiment 1. Short-term exposure to PM generated from candle burning; and Experiment 2. Short-term exposure to air pollution, including PM, due to commuting. In each experiment, the human subjects are tested under both elevated and ambient indoor PM concentrations. Both experiments have other characteristics that might affect cognitive performance, for example, commuting next to busy roads could heighten stress levels. In both experiments other air pollutants are released in addition to the PM. To test cognitive performance, three tests were used, these are: Mini-Mental State Examination (MMSE), Stroop color and word test - adult version, and Ruff 2 and 7 Selective Attention Test, which test the cognitive functioning, executive functioning, and visual attention (i.e. sustained attention, and selective attention), respectively. Detailed descriptions of these tests are provided in the methods section.

## Results

### Subject characteristics

The characteristics of the subjects studied in the two experiments were as follows. All subjects were healthy adults. Most subjects were under 24 years old and were predominantly students (73.3%, and 60.6% for experiment 1 and 2, respectively). Detailed information on the age and education demographics of the subjects, for the two experiments, is provided in Table 1.

**Table 1:** Characteristics of the study subjects.

	Experiment 1 (candle burning) n = 30	Experiment 2 (commuting) n = 33
Gender (male/female)	10/20	15/18
**Age %**		
Under 24 years	70	54.6
25–35 years old	16.7	21.2
36–45 years old	6.7	15.2
Over 56 years	6.7	9.1
Weight mean (SD)/kg	66.8 (16.1)	66.9 (14.9)
Height mean (SD)/cm	167 (10.7)	171 (9.6)
**Highest Level of Education %**		
-Secondary School	20	6.1
-High School	33.3	27.3
-UG degree/professional qualification	26.7	27.3
-Diploma/technical qualification	6.7	3
-PG degree	13.3	36.4
**Occupational position %**		
-Higher managerial, administrative and professional occupations	20	30.3
-Intermediate occupations	3.3	6.1
-Routine and manual occupations	3.3	3
-Student	73.3	60.6

### Experiment 1. Effect of candle burning on cognitive performance

30 subjects were tested in this experiment. A summary of the T-score results including mean, median, standard deviation (SD), Kolmogorov-Smirnov p-value, and t-test p-values are shown in Table 2. The T-score is the test score, which has been normalized for age and educational attainment of the subject, see methods. The average measured mass concentrations of PM_2.5_ (µg/m³) with and without candle burning are shown in Table 3.

**Table 2:** T-score results for cognitive tests performed in Experiment 1 (candle burning). Pre-exposure is in the absence of burning candles and post exposure is with burning candles present.

Test	Exposure time	Mean	SD*	median	K-S* p-value	t-test p-value
MMSE	Pre-exposure	47.9	15.9	56.0	>0.15	**0.011**
	Post-exposure	40.3	16.7	43.0		
Stroop Word	Pre-exposure	49.1	12.3	49.5	>0.15	0.652
	Post-exposure	48.3	14.0	51.5		
Stroop Color	Pre-exposure	50.4	8.6	51.5	>0.15	0.800
	Post-exposure	50.0	9.8	50.5		
Stroop Color-Word	Pre-exposure	58.7	8.9	58.5	0.096	0.658
	Post-exposure	59.3	9.4	59.0		
Stroop Interference	Pre-exposure	60.7	8.4	59.5	0.109	0.647
	Post-exposure	61.3	8.0	60.0		
Ruff 2 & 7 (Sustained attention-speed)	Pre-exposure	53.5	11.5	54.5	>0.15	0.628
	Post-exposure	52.9	12.1	52.5		
Ruff 2 & 7 (Sustained attention-accuracy)	Pre-exposure	47.0	10.6	51.0	>0.15	0.440
	Post-exposure	45.6	11.1	48.5		
Ruff 2&7 (Selective attention-ADS*)	Pre-exposure	52.5	10.7	53.0	>0.15	0.378
	Post-exposure	51.5	11.3	51.5		
Ruff 2 & 7 (Selective attention-ADA*)	Pre-exposure	47.8	10.1	51.5	0.045	0.228
	Post-exposure	45.7	10.4	49.5		
Ruff 2 & 7 (Selective attention-CSS*)	Pre-exposure	51.2	12.0	51.0	>0.15	0.623
	Post-exposure	50.6	12.3	51.0		
Ruff 2 & 7 (Selective attention-CSA*)	Pre-exposure	46.7	12.2	50.5	>0.15	0.862
	Post-exposure	46.3	13.2	50.0		

*K-S Kolmogorov-Smirnov; SD standard deviation; ADS Automatic detection speed; ADA Automatic detection accuracy; CSS Controlled search speed; CSA Controlled search accuracy. Significantly robust t-test p-values are emboldened.

**Table 3:** Average PM_2.5_ concentration during candle burning and without candle burning.

Exposure type	Mean ± (SD)	Median
PM_2.5_ Total Conc. (µg/m³) post-Exposure	41.4 ± (46.1)	27.0
PM_2.5_ Total Conc. (µg/m³) pre-Exposure	1.6 ± (1.3)	1.2

To test for differences between pre and post exposure T-scores for the different cognitive tests, the data sets were initially tested for normality using the Kolmogorov-Smirnov normality test. If the distributions were normal, then a two-sided paired t-test was performed comparing the mean of the pre-exposure and post-exposure T-scores. For all cognitive tests in experiment 1, the null hypotheses (H0) and alternative hypothesis (H1) listed below were used.

H0: Exposure to candle burning has no effect on cognitive performance (i.e. the mean scores are statistically equal).

H1: Exposure to candle burning has an effect on cognitive performance (i.e. the mean scores are not equal).

#### MMSE test

**Figure 1:**
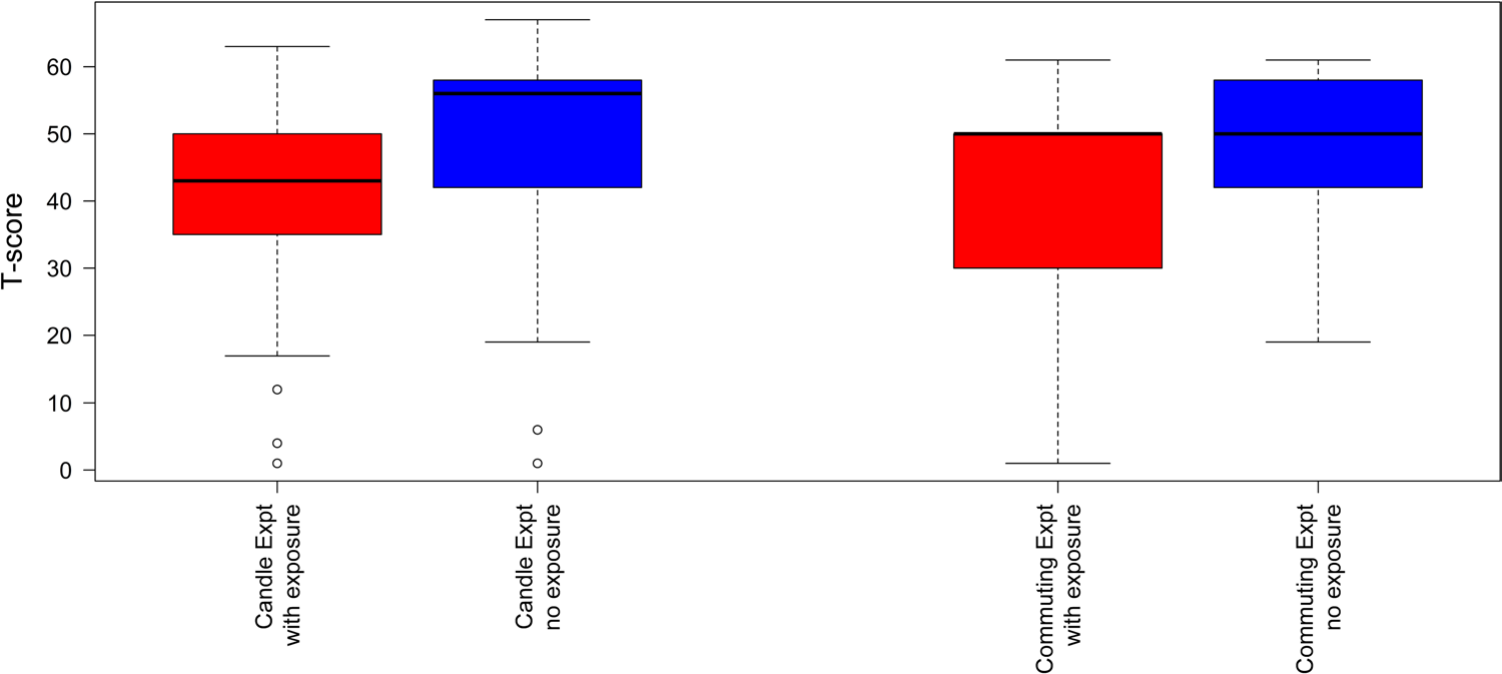
Box and whisker plots showing the MMSE T-score under exposure and non-exposure conditions for the candle burning and commuting experiments.

There was a large and statistically robust difference in the mean T-scores for the MMSE test with exposure to the burning candles reducing the mean T-score. Hence, short term exposure to candle burning has an adverse effect on short term cognitive performance. Box and whisker plots of the MMSE results are shown in Fig. 1, which clearly indicate significant differences in average cognition between pre and post exposure to candle burning.

The mean average T-scores for pre and post exposure to the candle burning were 48 ± 16 and 40 ± 17, respectively. The MMSE manual suggests a clinical interpretation of these scores as “average” and “below average” cognition, respectively.

**Figure 2:**
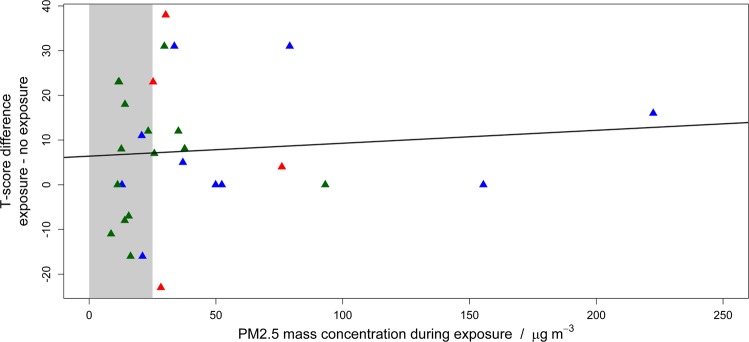
Comparison between MMSE T-score difference and PM_2.5_ mass concentration during exposure for the candle burning experiment. The difference values compare the post-exposure minus the pre-exposure values. Symbols represent type of candle: red – beeswax, blue – paraffin, green – stearin. The line represents the linear regression of the data points. Grey shaded area represents mass PM2.5 mass concentrations less than 25 µg m^−3^.

**Figure 3:**
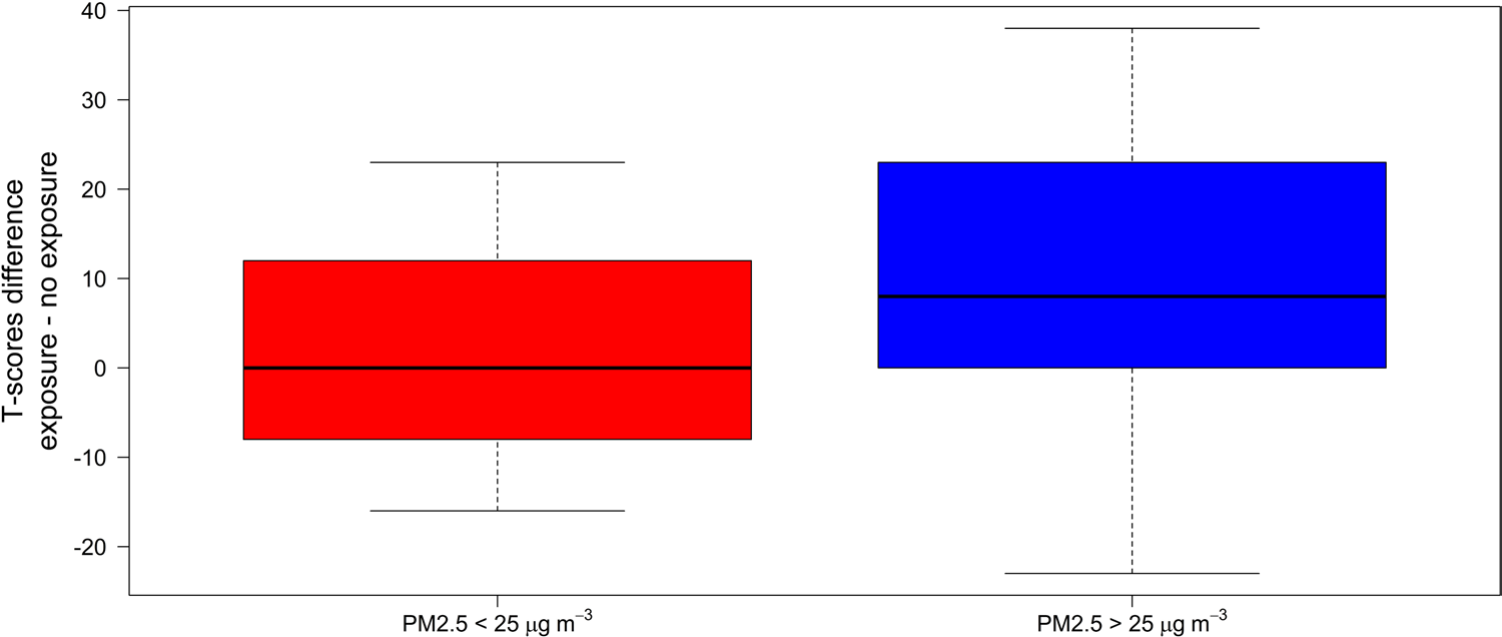
Differential MMSE T-score for the candle burning experiment when the PM_2.5_ mass concentration was above and below WHO recommendations for daily exposure (25 µg m^−3^).

To further investigate the effect of candle burning on cognitive performance, the effect of PM_2.5_ mass concentration upon cognitive performance was investigated. Figure 2 shows the effect of PM_2.5_ mass concentration upon cognition by plotting the differential T-scores (post exposure score minus pre exposure score) versus the differential PM_2.5_ mass concentrations (post minus pre exposure concentrations). There appears to be a tendency for subjects exposed to to the highest PM_2.5_ mass concentrations during the candle burning test to have a greater reduction in test performance after exposure to candle burning, however, a linear regression does not provide a statistically robust gradient value (p = 0.610). To further assess the apparent tendency, we compared the differential T-score to subjects who were exposed to a PM_2.5_ concentration above or below the daily WHO recommendation (25 µg/m³), see Fig. 3. The average differential T-score for when PM_2.5_ was significantly greater when the PM_2.5_ concentration is greater than the WHO recommendation compared to when it is less than the recommendation. The two distributions, with PM_2.5_ greater or less than the WHO recommendation, were not normally distributed, as assessed by the Kolmogorov-Smirnov normality test. Hence, the Mann-Whitney test was performed to compare the medians of the two groups different T-scores, the results showed that the p-value not adjusted for ties was 0.045, and adjusted for ties was 0.041. When the PM_2.5_ concentration was less than the WHO recommendation, the median differential T-score (=50) was significantly higher than the value obtained (=42) when the PM_2.5_ concentration was greater than the recommendation. This finding suggests that higher exposures to PM_2.5_ lead to a greater decline in short term cognitive performance. The seemingly non-linear relationship between cognition and PM_2.5_ concentration, see Fig. 2, suggests a threshold mass concentration of PM_2.5_ is required before cognitive decline is observed.

Three different candle types were used within Experiment 1, see methods. No statistically robust differences were observed between the uses of different candle types and measured cognitive scores, see Fig. 2.

#### Stroop Word-Color and Ruff 2 and 7 tests

Both the Stroop Word-Color test and the Ruff 2 and 7 test showed no significant difference (at the 5% significance level) between the T-scores measured for pre and post candle burning exposure. Hence no cognitive decline could be detected in the cognitive domains measured by these tests.

### Experiment 2. Effect of commuting upon cognitive performance

Two sided paired t-tests were performed to compare the mean pre-exposure and post-exposure scores of the cognitive tests with the following hypotheses:

H0: Exposure to pollutants from commuting has no effect on cognitive performance (i.e. the mean scores are equal).

H1: Exposure to pollutants from commuting has an effect on cognitive performance (i.e. the mean scores are not equal).

A summary of the results including mean, median, standard deviation (SD), Kolmogorov-Smirnov p-values, and t-test p-values are shown in Table 4. 33 subjects were tested in this project.

**Table 4:** T-scores results for cognitive tests from exposure to PM from commuting on cognitive performance before and after exposure.

Test	Exposure time	mean	SD*	median	K-S* p-value	t-test p-value
MMSE	Pre-exposure	49.6	9.5	50	0.02	**0.008**
	Post-exposure	41.9	15.9	50		
Stroop Word	Pre-exposure	44.6	12.4	45	0.031	0.391
	Post-exposure	47.1	12.2	48		
Stroop Color	Pre-exposure	47.1	12.3	45	>0.15	0.794
	Post-exposure	46.8	10.8	45		
Stroop Color-Word	Pre-exposure	55.9	14.4	55.9	>0.15	0.384
	Post-exposure	54.4	11.1	56		
Stroop Interference	Pre-exposure	60.1	9.0	59	>0.15	0.473
	Post-exposure	59.1	7.3	57		
Ruff 2 & 7 (Sustained attention-speed)	Pre-exposure	55.3	13.5	55	0.044	0.232
	Post-exposure	53.4	13.4	53		
Ruff 2 & 7 (Sustained attention-accuracy)	Pre-exposure	51.1	6.6	53	0.035	0.530
	Post-exposure	50.2	8.0	53		
Ruff 2 & 7 (Selective attention-ADS*)	Pre-exposure	56.2	13.2	54	>0.15	**0.006**
	Post-exposure	52.6	12.1	53		
Ruff 2 & 7 (Selective attention-ADA*)	Pre-exposure	51.9	3.8	52	0.047	0.634
	Post-exposure	51.4	6.0	53		
Ruff 2 & 7 (Selective attention-CSS*)	Pre-exposure	51.8	15.5	52	>0.15	0.300
	Post-exposure	50.3	15.2	50		
Ruff 2 & 7 (Selective attention-CSA*)	Pre-exposure	50.3	10.6	53	0.090	0.591
	Post-exposure	49.2	11.5	52		

*K-S: Kolmogorov-Smirnov; SD: standard deviation; ADS: Automatic detection speed; ADA: Automatic detection accuracy; CSS: Controlled search speed; CSA: Controlled search accuracy. Significantly robust t-test p-values are emboldened.

The pollutant concentrations in the commuting part of the project were not directly measured. However, an estimate of the outdoor air PM pollution can be obtained from the four Automatic Urban and Rural Network (AURN) monitoring sites which recorded the outdoor air quality in three locations in Birmingham^24^ (A4540 Roadside, Acocks Green, Tyburn). The average PM_2.5_ measurement during the experimental date and time period (14 February 2017–21 March 2017, 10:00 am–18:00 am) were 9.3, 9.8, 9.0 µg m^−3^, respectively, which are significantly higher than that measured in the pre-exposure testing in Experiment 1. It is noted; the AURN average PM_2.5_ concentration values likely provide a lower limit for the actual concentrations encountered by the commuters in Experiment 2 because of the greater distance of the AURN sites from the road compared to the commuters.

#### MMSE test

There was a large and statistically robust difference in the mean T-scores for the MMSE test with exposure to commuting reducing the mean T-score. Hence, this suggests short term exposure to commuting has an adverse effect on short term cognitive performance. Box and whisker plots of MMSE results are shown in Fig. 1. These results are similar to the results from the candle burning experiment.

The mean average T-scores for pre and post exposure to the commuting were 50 ± 9 and 42 ± 16, respectively. Similarly to the Experiment 1 outcomes, the MMSE manual suggests a clinical interpretation of these scores as “average” and “below average” cognition, respectively.

Comparison between the indoor ambient test scores in Experiments 1 and 2 revealed no difference in average cognition between the two cohorts tested in the different experiments.

#### Ruff 2 and 7 test

No cognitive decline was observed in any of the assessed domains except for selective attention - automatic detection speed, where the p-value was 0.006, providing very strong evidence for short term cognitive decline; this contrasted with the results for PM exposure from candle burning in experiment 1.

#### Stroop Word-Color test

The Stroop Word-Color test showed no significant difference (at the 5% significance level) between the T-scores measured for the pre and post commuting exposure. Hence no cognitive decline could be detected in the cognitive domains assessed by these tests.

## Conclusions and Implications

Decline in cognitive function can affect memory and attention, which can result in forgetfulness, inability to recall, and difficulty in decision making^21^. Cognitive decline is also closely linked to poorer mental health.

This is the first study to directly investigate the effect of PM exposure upon short-term cognitive performance in healthy adults. The results from the MMSE tests, which is a global assessment of an individual’s cognitive functioning, in both experiments show that short term cognitive decline occurs when a cohort is exposed to candle burning or commuting next to a major road. The decline in the measured T-scores, which are normalized for age and educational level, suggests that average cognitive ability of the cohorts, from both experiments, diminished from “average” to “low average” under the high PM exposure conditions.

Candle burning and commuting both increase personal exposure to PM air pollution, hence, it is hypothesized that PM pollution is the cause of the cognitive decline observed in both experiments. It is noted, both experiments have potential for causing cognitive decline through different mechanisms and future studies should strive to eliminate possible co-variables. The results from the Ruff 2 and 7 test, also indicated cognitive decline, in terms of selective attention, occurred when the cohort was exposed to commuting but not the candle experiment. However, the candle burning T-score did reduce after exposure to the high PM conditions, but not in a statistically significant manner. The Stroop test did not show any statistically robust change in measured cognition in either the candle burning or commuting experiment. It is perhaps surprising that it was the MMSE test, which provides a global assessment of cognitive function, provided statistically robust results in both the candle burning and commuting experiment, whereas the Ruff 2 and 7 and Stroop tests did not. Future work involving larger cohort sizes should now be attempted to see if the MMSE test results can be replicated in other cognitive tests.

This work is consistent with the study by Bos *et al*. (2013) study^25^, which examined the effect of aerobic training on cognitive performance in both rural and urban areas. The study suggested that the greater cognitive decline observed in the urban group could be due to air pollution being significant in cognitive performance. Bos *et al*. found that exposure to PM had potentially adverse effects upon cognitive performance in terms of executive function. It is noted, the Bos *et al*. study measured ultrafine particles (UFP, particles with a diameter less than 100 nm). Whilst UFP particles form a subset of the PM2.5 measured in this study, the sources and hence concentrations of UFP and PM_2.5_ are often distinct.

This study suggests important implications for human cognitive ability and mental health and their dependence upon PM exposure. It suggests that citizens of more polluted cities and countries will have, on average, worse cognitive ability than they would have if air quality was better. Therefore, this study suggests that reductions in PM air pollution will not only result in improved human morbidity and mortality outcomes but also upon cognitive performance. Further work is now required to better map out the effect of PM concentration upon cognitive performance. In the meantime, adherence to the WHO PM_2.5_ guidelines might ensure better mental health in addition to its aims of better physical population health.

## Methods

### Overall methodology

Two distinct human cohort experiments were conducted to establish whether short term exposure to air pollution has an effect on cognitive performance. The first experiment was designed to investigate whether the PM pollution from candle burning affected cognitive performance. The second investigated whether commuting on or close to major roads affected cognitive performance.

The criteria for participation in both projects were: healthy, non-smoking adults, English as a first language, non-occupationally exposed to air pollution, and not suffering from any medical conditions that could affect test performance (e.g. anxiety, colour blindness, fatigue, attention deficit disorder, etc.). 30 subjects were recruited for the first experiment, and 33 subjects were recruited for the second experiment. In the second experiment, three subjects did not have English as their first language and therefore were not tested for the Stroop color and word test. However, English as a first language is not a requirement for the other tests and hence they were tested for these. No subjects were involved in both experiments. Participation in the study resulted in a small financial reward of £30.

The experiments were advertised via posters placed at strategic locations within the University of Birmingham, UK. Postal adverts were also sent out to random addresses in the Selly Oak area of Birmingham, which is close to the University of Birmingham. Furthermore, an electronic announcement was posted to the University of Birmingham online student portal. Potential subjects responded by e-mail, or by contacting the office phone number. They were then sent further information about the research, including a participant information sheet, and a screening questionnaire with which to eliminate participants who did not meet the criteria of the various cognitive tests. Eligible subjects were then provided a consent form and a confounding factor questionnaire. The confounding factor questionnaire was completed by each candidate prior to each test (i.e. pre-exposure, then post-exposure), to check for any conditions that  might have affected their performance in the test. Subjects were informed that they could withdraw if they decided not to proceed with the study, and to facilitate this they were provided a withdrawal form in advance. None of the volunteers withdrew from the project.

Since both experiments required testing of participants twice, the experiments were investigated for order effects. The experimental design minimized order effects by pre-testing the subjects, as per test instructions. Investigation of the test scores subset by the order of the experiment (first versus second testing) revealed no statistically robust difference between the two subsets indicating the order of the testing is not significant.

Both projects gained full ethical approval from the Science, Technology, Engineering and Mathematics Ethical Review Committee (reference number ERN_16-0897) at the University of Birmingham. All methods were performed in accordance with the relevant guidelines and regulations. Informed consent was obtained from all participants.

### Materials

#### Cognitive tests and their description

All the instructions for taking the test, including the testing procedures, requirements, instructions given to the subjects, and scoring, were provided in the test manuals^26–28^. All the tests consisted of paper and pencil tests. Verbal instructions were given to the subjects before the tests, both for the pre-exposure and post-exposure testing. When subjects are tested successively, they may gain better results; therefore, the subjects had both pre-exposure and post-exposure tests not less than one day apart, to reduce the effect of practising. All the test scorings considered the age and education of the subjects.

### Mini-Mental state examination (MMSE)

This test is a global assessment of an individual’s cognitive functioning, including their memory, attention, orientation, and language, to indicate overall cognitive ability. The test consists of a set of questions and tasks which test the subject’s orientation to time, orientation to place, registration, attention and calculation, recall, naming, repetition, comprehension, reading, writing and drawing. The test consists of a booklet which is answered by the subject using a pencil.

Orientation to time: Questions about the year, season, month of the year, day of the week, and date, to assess the subject’s orientation to time.

Orientation to place: Questions about current place, to assess their orientation to place.

Registration: The subject is asked to repeat three words after the researcher says them, to assess their ability to learn and retain three unrelated words, and level of alertness and attentiveness.

Attention and calculation: Mathematical question about subtracting 7 from 100, then subtracting 7 from the answer, repeated four times (five answers in total).

Recall: The subject says the three words he/she repeated in the registration question, to assess their ability to recall the words learnt in the registration question.

Naming: Two questions to name any objects the researcher points to (such as pen, pencil, keys, etc.), to assess the subject’s ability to recognize and name two common objects.

Repetition: The subject is asked to repeat a sentence after the researcher says it, to assess the ability to repeat exactly a series of unrelated words that are not often said together.

Comprehension: The subject is asked to listen to and follow the researcher’s instructions to take a white paper with their right hand, fold it in half, and put it anywhere the researcher says, like on the table or the floor. This assesses their ability to attend to, understand and perform a complex three-stage command.

Reading: The test has a paper with the sentence “CLOSE YOUR EYES”. The researcher asks the subject to read and do what the paper says, to assess their ability to read and understand a simple sentence.

Writing: On a blank page, the subject should write a sentence that has both a subject and a verb, to test their ability to write a sentence.

Drawing: The test has a drawing of two intersecting pentagons, and the subject is asked to copy the design on a blank piece of paper, to assess their visuospatial ability.

The materials used for this test are the test booklet and a pencil^27^.

### Stroop color and word test - adult version

This test consists of three pages; each one has 100 items, presented in five columns of 20 items.

The first page is called the Word page, where the items are words written in black; these are “RED”, “BLUE”, and “GREEN”, arranged randomly. Here, the subject must read the words. The second page is called the Color page, which has colored items presented as XXXX written in either red, blue, or green. Here, the subject must say the color of the item. The third page is called the Color-Word page, that has colored words “RED”, “BLUE”, and “GREEN”, arranged randomly, written in either red, blue, or green ink. Here the subject must say the ink’s colour, not the word. For each page, the subject has to read the items out loud as fast as they can, starting from the top of the first column, and within the 45 seconds between the researcher saying “start” and “stop”. The test materials required for this test are the test booklet, pencil, and stopwatch.

The T-score for the “Word” page reflects the motor speech/reading sub-domain of cognition. The T-score of the “Color” page also reflects the subject’s motor speech in addition to intelligence, and the T-score for the color-word page is interpreted relative to the Color and Word scores, and is referred to the interference score. The interference T-score reflects the executive function; it does not necessarily indicate a problem with executive functioning if they have a low Color-Word score, as they could also have a low Word score, which might indicate they have a problem with reading, and therefore does not reflect executive function^28^.

### Ruff 2 and 7 selective attention test

This test measures two aspects of visual attention: sustained attention, and selective attention. Sustained attention is the ability to concentrate on one particular task, and maintain a consistent performance level over a continuous period of time, while ignoring distractors. Selective attention is the ability to select relevant targets while disregarding distractors^26,29–31^.

The test consists of a series of 20 trials (10 Automatic Detection trials and 10 Controlled Search trials). Each trial takes 15 seconds; hence, the total test takes five minutes. The subject should cross out all the 2 s and 7 s as quickly as possible, trying not to miss any, starting from left to right. They start over in the next series every 15 seconds when they hear the word ‘next’, until the five minutes are up, when the word ‘stop’ is heard. The materials used for this test are the test booklet, a stopwatch, and a brightly coloured pen, which enables easy detection of the outcome for the researcher.

Sustained attention is assessed by two variables: ‘total speed’, which is the total number of correct targets identified during the assigned five minutes duration; and ‘total accuracy’, which is the number of identified targets during the assigned five minutes’ duration divided by the number of possible targets^26,32^. Selective attention is assessed by two types of distractor conditions. The first is ‘automatic detection’, where the target digits, which are the numbers 2 and 7, are embedded in distractors which are alphabetic; it is called automatic because the numbers 2 and 7 are visibly and clearly a different stimulus category from the alphabetical distractors^26^. The second is the ‘controlled search’, where both targets (i.e. 2 and 7) and distractors are numbers and belong to the same stimuli category, so that selecting the target requires working memory involvement, which requires effort and is resource limited^26,33^.

#### Experiment 1. Effect of particulate matter emissions from candle burning on cognitive performance

Sample selection: After the screening questionnaire, potential subjects were given an information sheet to explain the project and their role, in addition to a meeting to answer further questions if they had any. After recruitment, subjects were given a consent form, which was to be signed by them, the researcher, and the supervisor, and a withdrawal form in case they no longer wanted to proceed with participation in the project.

Subjects performed the three cognitive tests in a quiet room with dimensions of 3.2 m × 3.1 m × 2.5 m, with the door and windows closed. For the pre-exposure test, the participants sat in the room with the windows and door closed for one hour. After one hour, the subjects performed the three cognitive tests. In the post-exposure test, the subject performed the tests after one hour exposure to PM_2.5_ produced from candle burning. All durations of exposure and non-exposure were uniform. Both pre and post exposure tests performed during similar times during working hours. A comfortable chair and desk were provided for the subject during the experiment. The pre-exposure test did not contain PM from candle burning but did contain ambient levels of PM which enter the room from outside through doors, windows and other small gaps. The post-exposure test included PM generated from candles and the ambient PM already present. The sources of the ambient PM are manifold and can be from outdoor emission sources including vehicles, construction work and natural sources^8^. In room sources could include the resuspension of dust by the subject and researcher walking in the carpeted room. To clear the air of candle related PM, the window was opened which allowed adequate ventilation for PM removal. To remove potential bias from the order of the pre and post-exposure testing, half the cohort took the pre-exposure test first and half took the post-exposure test first.

A 9-inch fan was used to homogenise the air composition within the study room; it was placed 75 cm away from the candles on a table. The table, on which the candles were placed, was obscured from the participants using a non-flammable insulation board so that the subject could not be visually aware of whether the candles were lit or not. Only one participant noticed and publicly asked whether candles were burning, they were not answered; this participant also mentioned that they had a high sensitivity to smells. No other subjects commented upon the presence, or not, of candles; however, this does not rule out the possibility that could detect differences between the pre and post-exposure tests.

PM mass was measured using an optical particle sizer instrument (TSI 3330); it is a portable light weight instrument that measures particle concentration (from 0 to 3,000 particles/cm^3^) in a range of particle size bins, with a maximum size of range of 0.3–10 μm, the reported size resolution is <5% at 0.5 μm. Particle size is estimated from the measured light scatter. Particle mass is estimated from the measured particle size. To convert from particle size to mass, a particle density of 1 g/cm^3^, and particle sphericity are assumed. The PM mass measured in this work is PM_2.5_, which is the mass of PM which has a diameter less than or equal to 2.5 μm in size. Once collected, the data are saved from the instrument using a USB stick, and downloaded by the Aerosol Instrument Manager® software. The software calculates the average PM_2.5_ mass observed during the test period.

The candles used varied in type (i.e. paraffin, beeswax, stearin) due to market availability, and all had a cotton wick. Different numbers of the same candle type were used in each test, because the PM concentrations vary from candle burning, and the concentrations are shown directly on the instrument’s screen.

A pilot experiment was conducted on one subject before the recruited subjects were tested. The pilot study ensured the test questions and confounding questionnaire were easily understood, and the room conditions were suitable for one hour of testing, i.e. it was possible for the subject to be comfortable, with no distractions.

#### Experiment 2. Effect of pollution from commuting on cognitive performance

The tests were performed in the same room as experiment 1. For the pre-exposure test, the subject sat in the room with the windows and door closed for one hour. After one hour, the subjects performed the three cognitive tests. In the post-exposure test, the subject performed the tests directly after commuting for approximately 30 minutes next to a major road either by walking, cycling, taking the bus or train. By commuting, the subjects were exposed to various traffic pollutants, including PM and gases including nitrogen oxides and carbon monoxide, which are known to affect health^10^.

### Statistical methodology and data analysis

The sample sizes were 30 in experiment 1, and 33 in experiment 2 for both MMSE and Ruff 2 and 7 tests, and 30 for Stroop Color and Word test; both sample sizes are sufficiently large to provide useful test results^34–36^.

The raw test scores for the three tests were converted to T-scores, which take into account age and education of the subjects. The procedure for conversion of the raw score to the T-score is provided in the test manuals.

Microsoft Excel 2016 was used to tabulate the results and provide descriptive statistics (mean, median, standard deviation). Minitab version 18 software was used to perform the statistical analyses. A Kolmogorov-Smirnov normality test was used to check data for normality, and a two-sided paired t-test was performed to compare the mean test scores for the pre-exposure and post-exposure tests.

The confounding questionnaire consists of 32 questions grouped into six parts, five concerning confounding factors that may affect test performance, and one part concerning socio-economic information. This information was not tested against the tests’ results because is lay outside the research objectives, but age and education were considered in scoring all the tests, and can be used in other papers and studies in future. Questions about confounding factors covered noise exposure, sleeping problems, emotional state, and caffeine consumption^37^.

## Supplementary information

supplementary information
