# Supplementary material for: Effects of short-term exposure to particulate matter air pollution on cognitive performance
Source: Sci Rep. 2019 Jun 3;9:8237. doi: 10.1038/s41598-019-44561-0 (PMC6546704; doi:10.1038/s41598-019-44561-0)
Supplement: Supplementary file 1 — supplementary information [file 41598_2019_44561_MOESM1_ESM.docx]

**Effects of short-term exposure to particulate matter air pollution on cognitive performance**

*M. A. Shehab and F. D. Pope*

*School of Geography, Earth and Environmental Sciences, University of Birmingham, Edgbaston, Birmingham, B15 2TT, UK.*

*Corresponding author* [*f.pope@bham.ac.uk*](mailto:f.pope@bham.ac.uk)

**Supplementary materials**

1. Screening Questionnaire
2. Confounding Questionnaire
3. **Screening Questionnaire**
4. Is English your first language?

Yes

No

1. What is your postcode? ------------------------------
2. Have you ever had brain surgery?

Yes

No

1. Have you ever had a brain injury?

Yes

No

1. Have you ever had an accident that affected your mental condition/ function and required you to visit the emergency room (ER)?

Yes

No

If yes, please specify what kind of accident (Car accident, work accident, bullet, assault...etc.)

------------------------------------------------------------------------------------------

1. Are you taking prescribed medication for any mental condition/ functions right now? (e.g. Memory problems, attention problems, judgment, recollection..etc.)

Yes

No

If yes, please describe the mental condition for which it has been prescribed, and what, if any are its side effects

Condition: ------------------------------

Side effects: ------------------------------

1. Have you ever been diagnosed with any of the following? (Circle all that apply)

Depression

Anxiety

Schizophrenia

Dementia

Attention deficit disorder

Fatigue

Multiple sclerosis (MS)

Brain cancer

Brain tumour

Other: -----------------------------------

1. Are you currently experiencing any of the following problems? (Circle all that apply):

Colour blindness

Headaches

Ringing in the ears

Dizziness

Irritability

Memory problems

Sleep problems

Concentration

Difficulty Problem Solving

Emotional changes

Changes in your relationships with others

Balance problems

Difficulty with reading, writing, calculating

Poor Judgment

Other: ------------------------------

**2. Confounding Questionnaire**

**Part one: Noise exposure**

- General information about noise in your everyday life

1. Are you exposed to loud noise...

... at your current home? ... at your current workplace?

Yes Yes

No No

If yes, please describe the source(s) of that noise and the amount of time you are exposed each day

Source:

Average hours per day: -----------------------------------------------------------------------

Average times per month: --------------------------------------------------------------------

Time: Day Evening

2. Do you regularly engage in noisy hobbies (e.g.: use of motorcycles, power tools, or loud music?

Yes

No

If yes, please describe:

3. Does the noise affect your sleep?

Yes

No

4. In which way does the noise affect your health?

- Information about noise in the 24-h prior to taking the test

5. Were you exposed to loud noise...

1. ... at your current home? b) ... at your current workplace? C)… somewhere else?

Yes Yes Yes

No No No

If yes, please describe the source(s) of that noise and the amount of time you were exposed in the 24-h prior to taking the test

Source:

6. Did the noise affect your sleep last night?

Yes

No

**Part two: Sleeping questions**

7. In general, do you have trouble ...

1. ... getting asleep? b) ... staying asleep?

Yes Yes

No No

8. Last night, did you have trouble...

1. ... getting asleep? b) ... staying asleep?

Yes Yes

No No

9. Do you usually wake up feeling refreshed on weekdays?

Yes

No

10. Did you wake up feeling refreshed this morning?

Yes

No

11. Do you feel you have a problem of any sort with your sleep?

Yes

No

If yes, please describe the problem:

12. How satisfied are you with the amount of sleep you get?

1. In general:

Dissatisfied Fair Satisfied

1. Last night:

Dissatisfied Fair Satisfied

13. Overall how would you rate the quality of your sleep?

1. In general:

Very poor Poor Fair Good Very good Excellent

1. Last night:

Very poor Poor Fair Good Very good Excellent

**Part three: Emotional State**

Please indicate how often each problem has bothered you during the past month and in the previous 24-h. Mark one of the boxes to the left that best corresponds to your problems:

14. Feelings of sadness

1. In general:

Not at all Seldom Sometimes Often All the time

1. During the last 24-h:

Not at all Seldom Sometimes Often All the time

15. Feeling easily irritated or annoyed

1. In general:

Not at all Seldom Sometimes Often All the time

1. During the last 24-h:

Not at all Seldom Sometimes Often All the time

16. Tension or inability to relax

1. In general:

Not at all Seldom Sometimes Often All the time

1. During the last 24-h:

Not at all Seldom Sometimes Often All the time

17. Diminished ability to think or concentrate

1. In general:

Not at all Seldom Sometimes Often All the time

1. During the last 24-h:

Not at all Seldom Sometimes Often All the time

18. Fatigue or loss of energy

1. In general:

Not at all Seldom Sometimes Often All the time

1. During the last 24-h:

Not at all Seldom Sometimes Often All the time

**Part four: General information about you**

1. Gender

Male Female

1. Age group

Under 24 years old

25-35 years old

36-45 years old

46-55 years old

Over 56 years old

1. What is your weight? ----------------
2. What is your height? ----------------

23. What is the highest degree or level of school you have completed? If currently enrolled, highest degree received

Secondary school

High school

Diploma/technical qualification

UG degree/professional qualification

PG degree

24. What is your present occupational position or (if no longer working) your last position?

Higher managerial, administrative and professional occupations

Intermediate occupations

Routine and manual occupations

Never worked and long-term unemployed

Student

25. What is your job title?

26. Your home. Mark one of the boxes to the left that best corresponds to your house:

- Do you own or rent your home?

27. How many people live in your home, including yourself? ----------------

28. How many bedrooms does your house have? ----------------

29. Do you have private health insurance?

Yes No

**Part five: Caffeine consumption:**

30. Do you generally consume caffeinated products? (e.g. tea, coffee, energy drinks, soft drinks, chocolate. etc.)

Yes No

31. When was the last time you had caffeine? ----------------

**Part six: Health status:**

32. Are you currently experiencing any of the following problems in the last 24 hours? (Circle all that apply):

Headaches

Ringing in the ears

Dizziness

Irritability

Memory problems

Sleep problems

Concentration

Difficulty Problem Solving

Emotional changes

Changes in your relationships with others

Balance problems

Difficulty with reading, writing, calculating

Poor Judgment

Other: ------------------------------
